# Drug Shop Intervention to Enhance Knowledge, Attitude, and Practices of Patent Medicine Vendors for the Control of COVID-19 In Southeastern Nigeria

**DOI:** 10.4269/ajtmh.20-1549

**Published:** 2021-06-15

**Authors:** Uchechukwu Madukaku Chukwuocha, Gregory Ndubeze Iwuoha, Onyeka Francis Ashinze, Princewill Ugochukwu Njoku, Chidera Chisom Obasi, Evelyn Ifezue Adey, Ikechukwu Nosike Simplicius Dozie

**Affiliations:** Department of Public Health, Federal University of Technology, Owerri, Imo State, Nigeria

## Abstract

Drug shops are the first point of care for most community members in low-resource countries. Because of symptomatic similarities with common illnesses such as malaria, probable coronavirus disease 2019 (COVID-19) cases may seek care at drug shops, where the knowledge and skills required to handle it may be lacking, thereby fostering community spread of the disease. This single-arm study provided an intervention to improve COVID-19-related knowledge, attitude, and practices of patent medicine vendors (PMVs) in 97 participating drug shops selected through cluster sampling in Owerri, southeastern Nigeria. The intervention involved a drug shop sensitization using information, education, and communication material, as well as training on the use of a risk assessment checklist to identify probable COVID-19 cases and to take appropriate action. Data were collected to determine the effect of this intervention using a pre-tested questionnaire and practice observation checklist, first at baseline and then 3 months post-intervention. Data analysis involved exploratory analysis and the *t*-test to determine pre- and post-intervention mean score differences at the 5% α level. There was post-intervention knowledge improvement on the COVID-19 causative pathogen (98.1% post-intervention versus 61.9% pre-intervention) and disease transmissibility from person to person (95.9% post-intervention versus 81.4% pre-intervention) among other knowledge domains. There was significant post-intervention improvement for positive attitude, with a mean gain score of 2.8 ± 1.7 (t = 4.4, *P* = 0.005), and preventive practices, with a mean gain score of 6.0 ± 4.7 (t = 4.1, *P* = 0.007). Engaging patent medicine vendors in the pandemic response plans through targeted interventions such as drug shop intervention could prove vital in the fight against COVID-19.

## INTRODUCTION

Coronavirus disease 2019 (COVID-19) is caused by severe acute respiratory syndrome coronavirus 2 (SARS-CoV-2), which spreads from person-to-person mainly through contact with respiratory droplets of an infected person.^1,2^ People infected with SARS-CoV-2 may be asymptomatic or present with flu-like symptoms such as cold, sore throat, headache, cough, fever, shortness of breath, fatigue, and pneumonia. It has been argued that the disease is more severe in the elderly than in young people.^3^ People burdened with underlying chronic diseases and the immunocompromised are also believed to be at greater risk of fatality.^4^ Because of the lack of a definite cure, and vaccines just emerging, combined with international travel and movement of goods and services, the disease spread quickly to all parts of the globe, including sub-Saharan Africa, which is home to many poor and underdeveloped countries.^5,6^ Nigeria’s first confirmed case of COVID-19 was reported on February 27, 2020; by April 2020, more than 9,500 cases, with 273 fatalities, were already confirmed.^7^ Since then, the disease has been spreading all over the country with increasing morbidity and mortality. According to the WHO, one of the core strategic responses to mitigating the effects of the pandemic is risk communication and community engagement.^8^ Already there have been several rumors and misconceptions in Nigeria that COVID-19 is a “white man’s” disease and does not affect black people, or people who have never been abroad.^9^ This kind of orientation can disrupt the implementation of interventions and adoption of safety guidelines, and may aid in fostering the spread of infection.

Engaging and enlightening the public on key issues bordering what is known and unknown about an outbreak, prevention guidelines, as well as new protocols are key to any emergency response. In various developing, underdeveloped, and resource-poor countries such as Nigeria, where the health and social infrastructures are weak, and affect the pattern of care-seeking negatively, private-sector drug shops are usually the first points of care for community members. They play an important role in delivering basic health services to the public^10,11^ and are operated by individuals designated as patent medicine vendors (PMVs). PMVs are drug distributors and sellers without formal pharmacy training who are licensed to retail proprietary and over-the-counter drugs, particularly for profit.^12^ They dominate most remote communities in Nigeria, including rural, suburban, and urban centers, and enjoy a certain level of respect and trust from community members. They are also generally regarded as a convenient source of relevant and correct health information.

Given that the symptoms of COVID-19 overlap those of common diseases such as malaria^12^ and the common cold, community members will most likely present with these same symptoms at private drug shops, where they usually obtain treatment. This makes drug shops potential points of infection and emphasizes the vital role of PMVs in the prevention of community transmission of COVID-19. It is therefore essential that these PMVs are adequately informed and trained through deliberate and targeted means to take necessary measures that will aid in curbing the community transmission of COVID-19, particularly from the drug shops. These measures include crowd management and physical distancing in the drug shop, use of gloves and face masks, frequent handwashing with soap and water, as well as disinfection with sanitizers after attending to customers. Other measures include the proper disposal of gloves and face masks after use, asking the right questions, educating the public, and following up by reporting suspected cases promptly to designated health authorities for appropriate action. Therefore, this study aimed at enhancing the knowledge, attitude, and practices of PMVs through a drug shop intervention to involve them in the control of COVID-19 in southeastern Nigeria.

## METHODS

### Study design.

This study was a single-arm intervention carried out between April and September 2020 to enhance the knowledge, attitude, and practices of PMVs by engaging and involving them in the control of the community transmission of COVID-19. Study participants were mobilized via engagement with the leadership of the National Association of Patent Medicine Vendors, Imo State Chapter, and subsequent text messaging in line with social and physical distancing guidelines.

### Study area.

The study was conducted in Owerri, Imo State, southeastern Nigeria. Imo State lies within latitude 4°45′N and 7°15′N, and longitude 6°50′E and 7°25′E, and measures 5,100 km^2^. The state has an estimated population of 4.8 million people and a population density that varies from 230 to 1,400 people/km^2^. Although there are a good number of health facilities in Imo, including hospitals and primary health-care centers, they are often either non-functional, ill-equipped, or understaffed.^13^ Private-sector drug shops dominate most parts of the state and supplement ideal health-care services in the area.

### Study population.

This study involved operators of private-sector drug shops in Owerri, registered with the Imo State Ministry of Health and the National Association of Patent Medicine Vendors. The drug shops totaled 904, serving about 1.4 million residents.

### Sample size estimation.

To estimate the study sample size (*n*), we considered the appropriate sample size computation technique at a 5% significant level:

$$n=\frac{p_{1}\times \left(100-p_{1}\right)+p_{2}\times \left(100-p_{2}\right)}{{\left(p_{2}-p_{1}\right)}^{2}}\times f\left(\alpha ,\beta \right),$$

where *p*_1_ and *p*_2_, respectively, represent known success rates for knowledge, attitude and practices (KAP) in the pre- and post-intervention groups, while α and β represent the type 1 error and the type 2 error respectively.

Because there were no prior studies in Nigeria on knowledge, attitude, and practices for the control of community transmission of COVID-19 among drug shops, we assumed level *p*_1_ = 50% and targeted a post-intervention improvement of 70% in 3 months (*p*_2_ = 70%). This led to a 20% minimum significant difference, at 80% power (*f* (α,β) = 7.9). A sample size of 91 drug shops was therefore estimated for the study. To account for the possibility of loss to follow-up, an additional 5% of the estimated sample was added, leading to a total number of 97 drug shops as the final estimated sample size.

### Sampling technique.

A two-stage cluster sampling technique was used to select the drug shops based on the probability proportional to the number of drug shops at each cluster of electoral wards in the study area. First, wards were selected through simple random sampling. This was followed by a systematic sampling of the required number of drug shops and their corresponding operators from each ward, who were then targeted for a physical engagement/interview.

### The intervention.

The intervention involved drug shop-to-drug-shop education and advocacy using information, education, and communication (IEC) material designed according to the guidelines of the WHO and the Nigerian Center for Disease Control (NCDC) on COVID-19. Furthermore, the drug shop operators were trained on probable case detection using a modified NCDC checklist. These activities were carried out during visits by two research team members per drug shop after they were updated adequately on the subject, protocols, and procedures. The contact time for each visit lasted an average of 1 hour. Study team members complied with all COVID-19 prevention guidelines and ensured these measures were also established at every drug shop visited during the intervention. The IEC material was a single page of material with four sections: 1) overview of COVID-19; 2) risk factors of COVID-19 in the drug shop, including factors that will enable community spread from the drug shop; 3) mitigation of risk factors to prevent community spread from the drug shop; and 4) actions to take on encountering a suspected case, including proper engagement of clients on the COVID-19 subject.

PMVs were also trained to use the modified NCDC checklist^7^ to extract information from clients who presented with symptoms associated with COVID-19. Questions on the form included experience of symptoms such as fever, headache, dry cough, and shortness of breath. Other questions were about recent visits to COVID-19 hot spots and contact with persons returning from COVID-19 hot spots. Each question on the checklist had a score. The total of the scores pulled according to the questions answered was expected to be calculated and compared with given thresholds to determine whether a client was a suspected case. If a case is suspected, the PMV is expected to counsel and extract the necessary information from the person, isolate the person in a safe, designated space in the drug shop, and report promptly to appropriate health authorities for extraction. The IEC material as well as copies of the checklist were made available to the PMVs for reference purposes and the determination of suspected cases. Telephone numbers of designated health authorities were also provided on the checklists for contact and reporting of suspected cases.

### Data collection.

Data were collected with the aid of a structured pre-tested questionnaire and a practice observation checklist. The suitability of the tools was assessed in a pre-test with 5% of the total sample size in drug shops located in another community close to the study area (Mbaitoli community). Data were collected twice: first at baseline (April 2020) before the intervention in May 2020, and then 3 months after intervention (August 2020). The data collection was done by four trained research assistants who were grouped in twos. One group administered the questionnaire while the other observed care practices in the drug shop and recorded their observations using the pre-tested observation checklist. Data collection lasted an average of 20 to 23 minutes at each site. The research assistants were not involved in administering the intervention.

### Data analysis.

Exploratory data analysis was performed initially, and the summary mean scores were computed for continuously recorded variables, whereas frequency distribution was formulated for distributional data. The summary of frequencies of the respective pre- and post-intervention responses for the knowledge and attitude domains as well as the recorded observations for the practices domain were used to assign scores ranging from 1 to 20, with 20 being the highest score. The mean of the respective pre- and post-intervention scores were calculated. The mean gain score was then determined as the difference between the post-intervention mean score and the pre-intervention mean score. A mean gain score of 1 represents 5% improvement in KAP. The effect of the intervention was established using a paired *t*-test at a 5% level. Statistical analysis was performed using Stata v. 14.1. (StataCorp, College Station, TX).

### Ethical approval.

Ethical approval for this study was sought and obtained from the ethical committee of the School of Health Technology, Federal University of Technology, Owerri, Nigeria, and the Imo State Ministry of Health, Owerri, Nigeria. Permission was also obtained from the Imo State chapter of the National Association of Patent Medicine Vendors. Informed written consent was obtained from all participating PMVs.

## RESULTS

### Characteristics of the study participants.

A total of 97 drug shops with the respective PMVs were involved in this study. The characteristics of the study participants are depicted in Table 1. About 54.6% were men and 45.4% were women, 54.6% were educated at the secondary school level, and 57.7% had more than 10 years’ experience in the business.

**Table 1:** Characteristics of the study participants (*N* = 97)

Characteristic	Frequency	Percent (%)
Gender		
Male	53	54.6
Female	44	45.4
Education Level		
Primary	4	4.1
Secondary	53	54.6
Tertiary	36	37.1
Postgraduate	4	4.1
Informal	0	0
Years of experience		
< 5	4	4.1
5–10	37	38.1
> 10	56	57.7

### Pre- and post-intervention introductory knowledge about COVID-19.

The study participants had good knowledge of the existence of, and pathological information relating to, COVID-19 Supplemental Table 1). All study participants had heard of COVID-19 prior to the intervention, with the majority (87.6%) knowing it resulted in a pandemic. Pre-intervention, only 61.9% were able to identify the pathogen responsible for COVID-19 as a virus. This, however, improved to 91.8% post-intervention. Knowledge about the lack of a definite cure for COVID-19 also improved from 41.2% (pre-intervention) to 77.3% (post-intervention).

**Table 2:** Pre- and post-intervention coronavirus disease 2019 knowledge classification (*N* = 97)

Knowledge	Pre-intervention			Post-intervention			*P* value	*t* Value
	Strong, *n* (%)	Fair, *n* (%)	Poor, *n* (%)	Strong, *n* (%)	Fair, *n* (%)	Poor, *n* (%)		
Introductory knowledge	51 (52.6)	35 (36.1)	11 (11.3)	89 (91.8)	8 (8.2)	0 (0.0)		
Symptoms, risk, and detection knowledge	61 (62.9)	22 (22.7)	14 (14.4)	69 (71.1)	17 (17.5)	11 (11.3)		
Knowledge on spread and transmissibility	76 (78.4)	21 (21.6)	0 (0.0)	97 (100.0)	0 (0.0)	0 (0.0)		
Preventive knowledge	65 (67.0)	24 (24.7)	8 (8.2)	97 (100.0)	0 (0.0)	0 (0.0)		
Overall knowledge	63 (64.9)	26 (26.8)	8 (8.2)	82 (84.5)	12 (12.4)	3 (3.1)		
Summary: Overall Good Knowledge								
Pre-intervention (mean ± SD, 14.00 ± 4.55)								
Post-intervention (mean ± SD, 18.00 ± 2.84)								
Gained score (mean ± SD, 4.00 ± 3.02)							0.001	5.939

Mean scores for coronavirus disease 2019 knowledge were determined using the summary of frequencies of the respective pre- and post-intervention responses to assign scores ranging from 1 to 20. Scores of 15 and greater were considered strong knowledge, scores of 10 to 14 were taken as fair knowledge, and scores less than 10 were considered poor knowledge.

### Pre- and post-intervention knowledge about symptoms, risk, and detection of COVID-19.

Supplemental Table 2 shows the pre- and post-intervention knowledge of studied PMVs about symptoms, risk, and detection of COVID-19. Pre-intervention, only 23.7% were able to identify more than 70% of the symptoms of COVID-19 whereas 43.3% were able to do so post-intervention. Knowing that everyone is at greater risk of COVID-19 also improved significantly post-intervention (94.8%) versus compared with pre-intervention (78.4%), as did knowledge about the standard test for confirming the disease (68% post-intervention versus 12.4% pre-intervention).

**Table 3:** Pre- and post-intervention attitude of studied patent medicine vendors toward coronavirus disease 2019 (*N* = 97)

Attitude	Pre-intervention		Post-intervention		*P* value	*t* Value
	Freq	%	Freq	%		
Does COVID-19 really exist?						
Yes	70	72.3	93	95.9		
No	15	15.5	0	0.0		
Don’t know	12	12.4	4	4.1		
Do you think you should be involved in efforts to control community spread of COVID-19?						
Yes	68	70.1	87	89.7		
No	29	29.8	10	10.3		
Do you think you are at risk operating your drug shop during this period?						
Yes	77	79.4	91	93.8		
No	20	20.6	6	6.2		
Do you support that infected persons should come to patent medicine vendors to purchase drugs for self-medication?						
Yes	12	12.4	4	4.1		
No	81	83.5	93	95.9		
Don’t know	4	4.1	0	0.0		
Should chloroquine/hydroxychloroquine be sold to customers when they complain of symptoms like those of COVID-19?						
Yes	29	29.9	8	8.2		
No	68	70.1	89	91.8		
Should patent medicine vendors educate their customers about COVID-19?						
Yes	93	95.9	97	100.0		
No	4	4.1	0	0.0		
Are you willing to embrace techniques to identify COVID-19 in case of reemergence or post-pandemic infections?						
Yes	85	87.6	97	100.0		
No	12	12.4	0	0.0		
Are you willing to adopt recommended precautionary measures while operating your shop during this period?						
Yes	69	71.1	71	73.2		
No	4	4.1	4	4.1		
Don’t know	24	24.7	22	22.7		
Overall positive attitude	75	77.3	89	91.8		
Pre-intervention (mean ± SD, 15.5 ± 1.92)						
Post-intervention (mean ± SD, 18.3 ± 1.74)						
Difference (mean ± SD, 2.8 ± 1.69)					0.005	4.388

COVID-19 = coronavirus disease 2019; Freq = frequency. Mean scores for attitudes toward COVID-19 were determined using the summary of frequencies of the respective pre- and post-intervention responses to assign scores ranging from 1 to 20. Scores up to 15 and above were considered as positive attitudes while scores below 15 were taken as negative attitudes.

### Pre- and post-intervention knowledge of spread and transmissibility of COVID-19.

Pre- and post-intervention knowledge of the spread and transmissibility of COVID-19 among studied PMVs is shown in Supplemental Table 3. The percentage of PMVs who knew that COVID-19 is transmissible from person-to-person improved from 81.4% (pre-intervention) to 95.9% (post-intervention). The knowledge that it spreads via respiratory droplets improved from 70.1% (pre-intervention) to 91.8% (post-intervention). All respondents affirmed fomite transmission of COVID-19 post-intervention (i.e., when one touches a contaminated surface and then touches the eyes, nose, and mouth without handwashing)—a shift from 91.8% pre-intervention.

**Table 4:** Pre- and post-intervention coronavirus disease 2019 control-related practices among studied patent medicine vendors (*N* = 97)

Practice	Pre-intervention		Post-intervention		*P* value	*t* Value
	Freq	%	Freq	%		
Handwashing facilities provided in the drug shops						
Running tap water with soap	12	12.4	12	12.4		
Running bucket water with soap	24	24.7	81	73.5		
Basin stagnant water with soap	0	0.0	0	0.0		
Hand sanitizers only	21	21.6	8	8.2		
None	28	28.9	0	0.0		
Thermometer used to read body temperature of customers at the drug shop						
Infrared thermometer	20	20.6	57	58.8		
Ordinary clinical thermometer	8	8.2	8	8.2		
None	69	71.1	32	33.0		
Other	0	0.0	0	0.0	
Strict enforcement of physical distancing among customers in the drug shop						
Yes	52	53.6	89	91.8		
No	45	46.4	8	8.2		
Prohibition of customers from touching surfaces or objects in the drug shop						
Yes	56	57.7	89	91.8		
No	41	42.3	8	8.2		
Keeping at least 2 m of physical distancing when attending to customers						
Yes	60	61.9	89	91.8		
No	37	38.1	8	8.2		
Method of exchanging pleasantries with customers						
Oral greeting/waving hands	64	66.0	85	87.6		
Handshakes	21	21.6	4	4.1		
Touching of arms or elbows	12	12.3	8	8.2		
Bowing	4	4.1	0	0.0		
Other	0	0.0	0	0.0	
Frequent wiping of surfaces and high-contact areas with alcohol-based sanitizers						
Yes	89	91.8	97	100.0		
No	8	8.2	0	0.0		
Practice of good personal hygiene in the shop						
Yes	97	100.0	97	100.0		
No	0	0.0	0	0.0	
Wearing of gloves while administering first aid to customers						
Yes	85	87.6	93	95.9		
No	12	12.3	4	4.1		
Practices when customers present with symptoms that resemble symptoms of COVID-19 (fever, cough, difficulty breathing)						
Administer normal drugs	49	50.5	0	0.0		
Send away	12	12.4	4	4.1		
Use the NCDC self-checklist for case detection	28	28.9	93	95.9		
Other	4	4.1	0	0.0		
Action when a customer scores between 12 and 28 on the NCDC checklist						
Panic and send away	4	4.1	4	4.1		
Counsel and call appropriate health authorities	28	28.9	89	91.8		
Refer to hospital	8	8.2	4	4.1		
Refer to herbalist	45	46.4	0	0.0		
Counsel and advise to contact health authorities	8	8.2	0	0.0		
Other (administer some drugs and send away)	8	8.2	0	0.0		
Provision of COVID-19-related information to customers						
Yes	68	70.1	97	100.0		
No	29	29.9	0	0.0		
Willingness to report suspected cases to the designated authorities						
Yes	24	24.7	84	86.6		
No	69	71.1	13	13.4		
Don’t know	4	4.1	0	0.0		
Provision of isolation point around the shop where a suspected case could be kept until designated authorities arrive						
Yes	0	0.0	4	4.1		
No	97	100.0	93	95.9		
Sell of COVID-19-related personal protective equipment such as mask, gloves, hand sanitizers, and others to customers						
Yes	77	79.4	81	83.5		
No	20	20.6	16	16.5		
Overall practice (good practice)	58	59.8	87	89.7		
Pre-intervention (mean ± SD, 12.06 ± 5.59)						
Post-intervention (mean ± SD, 18.04 ± 1.91)						
Gained score (mean ± SD, 5.98 ± 4.70)					0.007	4.059

COVID-19 = coronavirus disease 2019; Freq = frequency; NCDC = Nigerian Center for Disease Control. Multiple responses considered. Mean scores for COVID-19-related practices were determined using the summary of frequencies of the respective pre- and post-intervention recorded observations to assign scores ranging from 1 to 20. Scores of 15 and greater were considered good practice, whereas scores less than 15 were taken as poor practice.

### Pre- and post-intervention knowledge of COVID-19 preventive measures.

A majority of the respondents showed reasonable knowledge about COVID-19 preventive measures, especially post-intervention (Supplemental Table 4). Pre- and post-intervention knowledge about COVID-19 preventive measures included the practice of social distancing (66.0% pre-intervention versus 93.8% post-intervention), frequent handwashing with soap and water (91.8% pre-intervention versus 95.9% post-intervention), and wearing of face masks and gloves (91.8% pre-intervention versus 95.9 post-intervention). Respondents also knew the required physical distance in the drug shop (37.1 pre-intervention versus 79.4 post-intervention), and that isolation and prompt treatment of infected persons are effective ways of limiting the spread of COVID-19 (79.4% pre-intervention versus 100% post-intervention).

### Pre- and post-intervention classification of COVID-19 knowledge among PMVs.

Strong knowledge about COVID-19 spread and transmissibility as well as preventive practices improved to 100% post-intervention from 78.4% and 67%, respectively, pre-intervention (Table 2). Pre-intervention-to-post-intervention improvements were also recorded in the following knowledge domains: introductory knowledge of the disease (52.6–91.8%), and symptoms, risk, and detection (62.9–71.1%).

### Pre- and post-intervention attitude of studied PMVs toward COVID-19.

Table 3 depicts improvement in positive attitude of studied PMVs toward COVID-19, where 72.3% pre-intervention and 95.0% post-intervention agreed that COVID-19 exists in Nigeria. About 83.5% of PMVs pre-intervention and 95.9% of PMVs post-intervention agreed that COVID-19-infected persons should not go to drug shops to purchase drugs for self-medication. Furthermore, 95.9% pre-intervention versus 100% post-intervention agreed that they should educate their customers about COVID-19.

### Pre- and post-intervention COVID-19 control-related practices among studied PMVs.

Significant improvements in COVID-19-related good practices were observed, as depicted in Supplemental Table 4. The proportion of drug shops that provided running water using a suspended bucket with tap and soap for handwashing increased from 24.7% pre-intervention to 73.5% post-intervention. The percentage of PMVs who did not check their customers’ temperature using any type of thermometer reduced from 71.1% pre-intervention to 33% post-intervention. PMVs who were observed to enforce physical distancing among their customers increased from 53.6% pre-intervention to 91.8% post-intervention. The percentage of studied PMVs who used the NCDC checklist to score and counsel customers also increased, from 28.9% pre-intervention to 91.8% post-intervention. More drug shop operators provided COVID-19-related information to their customers post-intervention (100%) versus pre-intervention (70.1%), and more PMVs (86.6%) post-intervention became willing to report suspected cases to the designated response team, compared with 24.5% pre-intervention.

## DISCUSSION

In most parts of Nigeria, drug shops are often the first points of care for persons with mild and moderate illnesses with symptoms similar to those of COVID-19, and generally play a significant role in the Nigerian health-care cascade. A failure to integrate drug shops into COVID-19-related strategic response plans may create significant bottlenecks in efforts to control the disease, particularly at the community and household levels. Training and sensitizing PMVs on recent developments in health, especially during epidemics/pandemics such as that caused by COVID-19, might prove key in breaking the chain of infection in various rural and semi-urban settings. Because of the novelty of SARS-CoV-2, there has been no defined cure or vaccine for COVID-19 until recently. This emphasizes the need to maximize the use of every available resource in efforts to control the disease. PMVs hold certain key elements in unlocking the minds of community members to adhering to guidelines, rules, and regulations that have been set by the government and appropriate authorities to aid in the fight against COVID-19. Some of the key elements are that PMVs understand the communities in which they operate, command respect, influence the people, and are familiar with the leaders of their communities. These factors make it easy for them to convey, potentially, vital information concerning COVID-19 to community members and remind them of their responsibilities. This is because it is only when people accept and regard a source of information as trustworthy, can they begin to abide by the information.

Our study shows the PMVs were relatively knowledgeable about COVID-19 both pre- and post-intervention. Studies on COVID-19 KAP in parts of north–central Nigeria,^14^ and among health-care workers^15^ and senior pharmacy students^16^ all reported that respondents had good general knowledge about the disease. Our drug shop intervention, however, improved this general knowledge significantly among PMVs, particularly on specific COVID-19 knowledge domains, including knowledge about symptoms, risky behaviors, detection and diagnosis, spread and transmissibility, as well as knowledge about preventive measures. Improvements in the domains of symptom recognition, identification of risky behaviors, detection, and diagnostic procedures as well as the transmissibility of COVID-19 were quite remarkable. These changes may have been triggered by the level of consciousness created by the disease, which made the intervention appealing and acceptable to the participants; hence, they were enthusiastic to learn more. The knowledge level among participants was not a surprise given that lots of COVID-19-related information was circulating the Internet and social media, although some of it may not be reliable resulting from a lack of control. Other sources of information such as radio and television could have also played a part in this. Nevertheless, the participants’ perception that the disease has a definite cure poses a major concern because it can easily be a reflection of the general public’s perception, such as unconfirmed information that hydroxychloroquine was a definite and recommended cure for COVID-19.

There was a significant improvement in positive attitude regarding COVID-19 post-intervention, implying the potential sustainability of the intervention. A positive attitude is important and paramount to ensuring that interventions work, and that people accept, trust, and live based on the information they receive from authorities.^17^ Interestingly, a reasonable number of the studied PMVs believed that COVID-19 exists in Nigeria, despite the misleading news at the time that COVID-19 was not in Nigeria and that the government was taking advantage of the global menace to embezzle money. Perhaps this is attributable to the fact that the PMVs have a slight edge over the lay public on how they perceive health-related challenges, given their line of business. Because these PMVs are often in steady contact with the public, they can help correct prevailing misconceptions and misinformation, especially if validated and encouraged to do so. Regrettably, only a few of them expressed satisfaction with measures put in place so far to contain the virus even after our intervention. This corroborates the report of another Nigerian study in which most of the respondents expressed dissatisfaction with the measures set by the government and appropriate authorities to control COVID-19.^14^ Our study participants, however, expressed their willingness to take measures against the spread of COVID-19 regardless of what the government had put in place. This suggests there could be a disconnect with the authorities that may need to be addressed properly so that everyone is onboard with ensuring that COVID-19 is controlled effectively in Nigeria. Significant improvement in terms of good practices was recorded with the intervention, as more PMVs post-intervention were inclined to use face masks and gloves, and encouraged their customers to practice physical distancing, hand washing, and good personal hygiene. This improvement was also evident in the number of drug shops post-intervention with improvised running water using a suspended bucket with a tap and bowl beneath to collect the runoff water. Other practices observed to have improved in the drug shops post-intervention were enforcement of physical distancing, discouragement of customers from touching high-contact surfaces, sanitization of surfaces, and non-contact greetings. These are some of the highly recommended measures to limit the spread of COVID-19.^18^ Although the effective use of face masks and gloves is key to mitigating the spread of COVID-19 in the drug shops, attention needs to be paid to their quality to ensure their durability and effectiveness. For instance, some of the face masks worn by some of the respondents appeared overused, dirty, and may have been contaminated, and some PMVs were not quite sure of the alcohol content in the hand sanitizers they used. In China, the Quality Control Center once reported that many health-care workers at Hubei Province were infected with COVID-19 because of the improper use of personal protective equipment, including face masks and gloves.^19^ Likewise, another study in southwest and northwest Nigeria reported that only 25.7% of the study group had adequate knowledge about personal protective equipment.

Along with improvement in the rate of customer sensitization, our intervention also contributed to an increase in the rate of the use of the NCDC COVID-19 recommended checklist for case detection at the drug shops studied. Although no COVID-19 cases were reportedly confirmed from any of the shops during the study period, this is a critical aspect of the intervention that needs to be sustained because the PMVs’ ability to counter further spread of COVID-19 in the community hinges on their capacity to identify and report suspected cases promptly. On the other hand, there was no significant change in the provision of a temporary isolation point at the drug shops where suspected cases may remain until the designated authorities arrive. This could have been a result of the lack of space rather than willingness to make such an area possible. The majority of the structures housing these businesses are small buildings situated along local roads. This poses a great concern, especially as response from the designated authorities may not be as prompt as it should be. Strengthening the capacity for case-finding, case detection, isolation, and prompt response is key to reducing the spread of COVID-19.^20^

The post-intervention behaviors recorded in our study can be sustained by adopting and integrating the intervention as part of the national COVID-19 control activities in which compliance with the recommended practices can be enforced by regulatory agencies. Text messaging could also be used to remind PMVs of these practices and sustain positive behavior. Furthermore, visual aids, such as posters displayed at strategic points in the drug shops, could serve as constant reminders for PMVs to adhere to the recommended, appropriate COVID-19-related care practices.

### Limitations.

A limitation of this study included our inability to assess the extent to which drug shop operators were able to influence their customers and or community members positively to adopt and maintain good practices that disrupt the spread of infection. In addition, because of the relatively small population size and the intent to engage as many drug shops as possible, we could not introduce a control group; hence, a single-arm intervention design was adopted. While the intervention was being carried out, general public education on COVID-19 was in place, which may have contributed to the changes recorded post-intervention. However, an intervention such as this is important in delivering targeted and relevant information to ensure adequate awareness and appropriate practices among relevant groups such as the PMVs.

## CONCLUSION

The level of health knowledge along with other significant factors play a major role in determining health behavior and how interventions are accepted and used. With novel infections such as COVID-19, communicating relevant, correct information to the public may help facilitate control and mitigation efforts. The findings of our study affirm that massive enlightenment as well as a positive attitude and good practices toward COVID-19 can be achieved through the introduction of interventions that strengthen community sensitization by including private-sector health-care providers in the fight against the disease. Organizing training for drug shop operators and similar players in the health-care system of Nigeria should always be considered. This is because it may help create a more universal approach to responding to emergencies caused by contagious diseases and may even improve the quality of services provided at the drug shops. This measure is evidently needed in Nigeria and other developing nations, where most health services are provided primarily outside government-owned facilities and most are financed through out-of-pocket spending. Furthermore, the engagement and integration of these critical community health stakeholders are of additional value during post-pandemic surveillance to ensure that residual cases are detected and handled promptly and adequately. Drug shop operators could also play important roles in the enlightenment of community members regarding COVID-19 issues, including sensitization and mobilization for vaccinations. Failure to engage and involve critical stakeholders in community health-care delivery, such as PMVs, may foster community transmission of COVID-19 and exacerbate the pressure on the already weak health infrastructure to meet the health demands of the people.

This study has demonstrated the drug shop intervention to be effective in enhancing the KAP of PMVs in engaging them in efforts to contain COVID-19. It has the potential for sustainability and scalability because of its acceptance, cost-effectiveness, simplicity, and ease of implementation. It is, however, necessary to carry out large-scale studies to explore its real impact in combating the pandemic, particularly in sub-Saharan Africa and other low-resource countries.
